# Continuous-Flow High-Pressure Homogenization of Blueberry Juice Enhances Anthocyanin and Ascorbic Acid Stability during Cold Storage

**DOI:** 10.1021/acs.jafc.4c01289

**Published:** 2024-05-13

**Authors:** Jayashan Adhikari, Lida Rahimi Araghi, Rakesh Singh, Koushik Adhikari, Bhimanagouda S. Patil

**Affiliations:** † Vegetable and Fruit Improvement Center, Department of Horticultural Sciences, Texas A&M University, 1500 Research Parkway, Suite A120, College Station, Texas 77845-2119, United States; ‡ Department of Food Science and Technology, University of Georgia, 100 Cedar Street, Athens, Georgia 30602, United States; § Department of Food Science and Technology, University of Georgia, 1109 Experiment Street, Griffin, Georgia 30223, United States; △ Department of Food Science and Technology, Texas A&M University, 1500 Research Parkway, Suite A120, College Station, Texas 77845-2119, United States

**Keywords:** preservation, bioactive compounds, polyphenol oxidase, interplay, quality retention

## Abstract

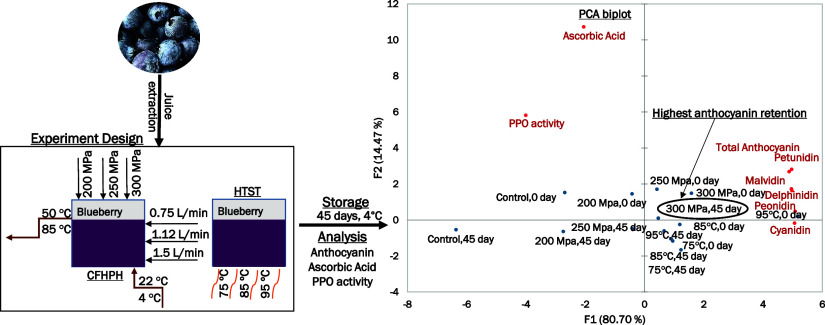

Blueberries (*Vaccinium* section *Cyanococcus*) have a wealth of bioactive compounds, including anthocyanins and other antioxidants, that offer significant health benefits. Preserving these compounds and maintaining the sensory and nutritional qualities of blueberry products such as juice during cold market storage is critical to meet consumer expectations for nutritious, safe, and minimally processed food. In this study, we compared the effects of two preservation processing techniques, high-temperature short-time (HTST) and continuous flow high-pressure homogenization (CFHPH), on blueberry juice quality during storage at 4 °C. Our findings revealed that inlet temperature (*T*_in_) of CFHPH processing at 4 °C favored anthocyanin retention, whereas *T*_in_ at 22 °C favored ascorbic acid retention. After 45 days of storage, CFHPH (300 MPa, 1.5 L/min, 4 °C) juice retained up to 54% more anthocyanins compared to control at 0 day. In contrast, HTST treatment (95 °C, 15 s) initially increased anthocyanin concentrations but led to their subsequent degradation over time, while also significantly degrading ascorbic acid. Furthermore, CFHPH (300 MPa, 4 °C) juice had significantly lower polyphenol oxidase activity (>80% less than control), contributing to the overall quality of the juice. This innovative processing technique has the potential to improve commercial blueberry juice, and help meet the rising demand for healthy and appealing food choices.

## 1 Introduction

Blueberries (*Vaccinium* section *Cyanococcus*) are delectable and nutritious berries with sweet and tart flavors, blue color, and numerous health benefits.^1^ Anthocyanins, a type of flavonoids, are water-soluble pigments that give blueberry its characteristic red-blue color and have various health-beneficial properties.^2^ Blueberries contain a diverse array of anthocyanin glycosides, including glucosides, galactosides, and arabinosides, and minor quantities of acylated derivates of delphinidin, cyanidin, petunidin, peonidin, and malvidin.^3^ Furthermore, they are rich in vitamins, particularly the essential vitamins A, C, E, and K, with vitamin C (ascorbic acid) present in high concentrations.^4^ A diet rich in blueberries is linked to a healthier lifestyle, attributed to their abundant content of anthocyanins and ascorbic acid. Anthocyanins, as widespread natural pigments, demonstrate health benefits through modulation of various signaling pathways and cellular processes, potentially serving as therapeutic targets for a range of diseases.^5^ Similarly, ascorbic acid, known for its antioxidant and anti-inflammatory properties, not only scavenges free radicals but also activates intracellular antioxidant systems, highlighting its potential to prevent oxidative damage and inflammation in cells.^6^

The increased awareness of the nutritional and health-promoting benefits associated with the consumption of blueberries has enhanced the demand for fresh as well as processed blueberries, resulting in enhanced cultivation and consumption of blueberries every year. Farmers in the United States cultivated ∼280,000 t of blueberries in 2022, of which ∼130,000 t was processed to meet the growing demand for blueberry products.^7^ The seasonal availability and perishable nature of blueberries have prompted the need for efficient processing and preservation methods. However, for blueberry juice, traditional approaches such as thermal pasteurization and chemical preservation often have adverse effects on anthocyanins and vitamins, thereby diminishing the overall health benefits and quality of juice over time.^8−10^ For example, Patras et al.^11^ described studies showing that thermal processing led to a significant reduction in the content of phenolic compounds and anthocyanins, which are important contributors to the antioxidant capacity of the juice. Additionally, thermal processing led to a reduction in the sensory quality of the juice, including a decrease in the color intensity and overall acceptability. Other processing-related problems include color changes, oxidation, and the growth of microbes during storage.^12^ Anthocyanins are sensitive to temperature and environmental conditions and thus can be used as markers to assess product quality during processing and storage.^13^

Various pre- and postharvest processing and preservation methods can affect the nutrient quality and quantity of fruit juices during processing.^11,14−16^ For example, heating methods such as pasteurization and hydrothermodynamic processing can damage anthocyanins and create unwanted polymeric byproducts.^11,17^ Therefore, the food industry has been employing several methods to ensure the safety and stability of fruit products, and nonthermal processing techniques like pulse electric field (PEF), ultrasound, high-pressure processing, and homogenization have gained popularity due to the increasing demand for fresh-like juice products.^18−20^ Various research studies have shown that high-pressure homogenization (HPH) is an effective method for reducing microbial load, inactivating degradative enzymes, and preserving the nutritional and sensory properties of foods, making it a promising technique for processing and preservation.^21−23^ In our previous study, Megatron and high-pressure homogenization (HPH) were employed to produce micronized tart cherry purée, resulting in a higher bioavailability of antioxidants.^24^ The HPH samples showed an increase in the yield of polyphenol extraction two times that of the nonmicronized samples. However, HPH is a batch processing, which has higher unit costs.^25^

Therefore, we introduce continuous flow high pressure homogenization (CFHPH), which offers the advantage of enhanced liquid flow and large-scale production, overcoming the limitations of batch processing. The process involves pumping juice through a narrow valve under high pressure, typically from 100 to 400 MPa, causing the particles to break down into smaller sizes due to cavitation, shear forces, and turbulence. After the pressure is released, the smaller particles recombine in a more uniform distribution, resulting in stable emulsions and improved texture and consistency of final products.^21,26,27^ Although CFHPH and HPH work on the same principle, CFHPH involves the continuous impact of the pressure, flow rate, inlet temperature, and residence time on the juice quality. There’s a research gap regarding CFHPH and its impact on the nutritional quality of fruit juice compared to conventional thermal processing. Moreover, there is no promising research on the effects of continuous flow high-pressure homogenization on blueberry juice and its overall effect on the nutritional composition during refrigerated storage. It is crucial to assess the comparability of these processing methods, especially in preserving thermally sensitive phytochemicals like anthocyanins and vitamin C.

In this study, we evaluated the effects of CFHPH on blueberry juice processing at various levels of pressure, flow rate, and inlet temperature and assessed the impact on anthocyanins, vitamin C contents, and polyphenol oxidase (PPO) activity during refrigerated storage. By evaluating the effects of CFHPH on blueberry juice preservation processing, this study can help determine the feasibility of CFHPH as a viable alternative for preserving the nutritional properties and shelf life of fruit juices.

## 2 Materials and Methods

### 2.1 Chemicals and Reagents

Delphinidin-6-glucoside (purity grade ≥97%), cyanidin-6-glucoside (purity grade ≥99%), petunidin-6-glucoside (purity grade ≥99%), malvidin-6-glucoside (purity grade ≥99%), peonidin-6-glucoside (purity grade ≥95%), pyrocatechol (purity grade >99%), and l-ascorbic acid (purity grade ≥99%) were purchased as standard compounds from Sigma-Aldrich (St. Louis, MO, USA). HPLC-grade methanol, formic acid, acetonitrile, and metaphosphoric acid were purchased from Sigma-Aldrich (St. Louis, MO, USA). Pectinase enzyme (Pectinex Ultra SP-L) was procured from Novozymes A/S, Switzerland, and distributed by Sigma-Aldrich (St. Louis, MO, USA). HPLC-grade water (resistivity 18.2 mΩ cm) was obtained from a Nanopure water purification system (Barnstead, Dubuque, IA, USA). All other chemicals are of HPLC grade.

### 2.2 Blueberry Juice Processing

#### 2.2.1 Blueberry Sample Collection and Juice Preparation

Frozen Brightwell rabbiteye blueberries (*Vaccinium virgatum*, grown in Georgia) were bought from Farmer John LLC. (Alma, Georgia, USA), and stored at −40 °C before processing. Blueberries were thawed overnight at 10 °C, and the pulp was prepared using a juice blender (Hobart Corp. Troy, OH, USA). To ensure effective depectinization of the pulp without negatively impacting the sensory properties and nutritional value of fruit juices, a concentration of 0.00827% (v/v) pectinase enzyme was added to the pulp and kept at 35 °C for 1 h, as employed in prior studies.^28,29^ The pulp was then pressed using a bladder press (Willmes GmbH, Lorsch, Germany) and filtered using six layers of grade 90 cheesecloth to form a blueberry juice sample.

#### 2.2.2 Continuous Flow High Pressure Homogenization

**Figure 1:**
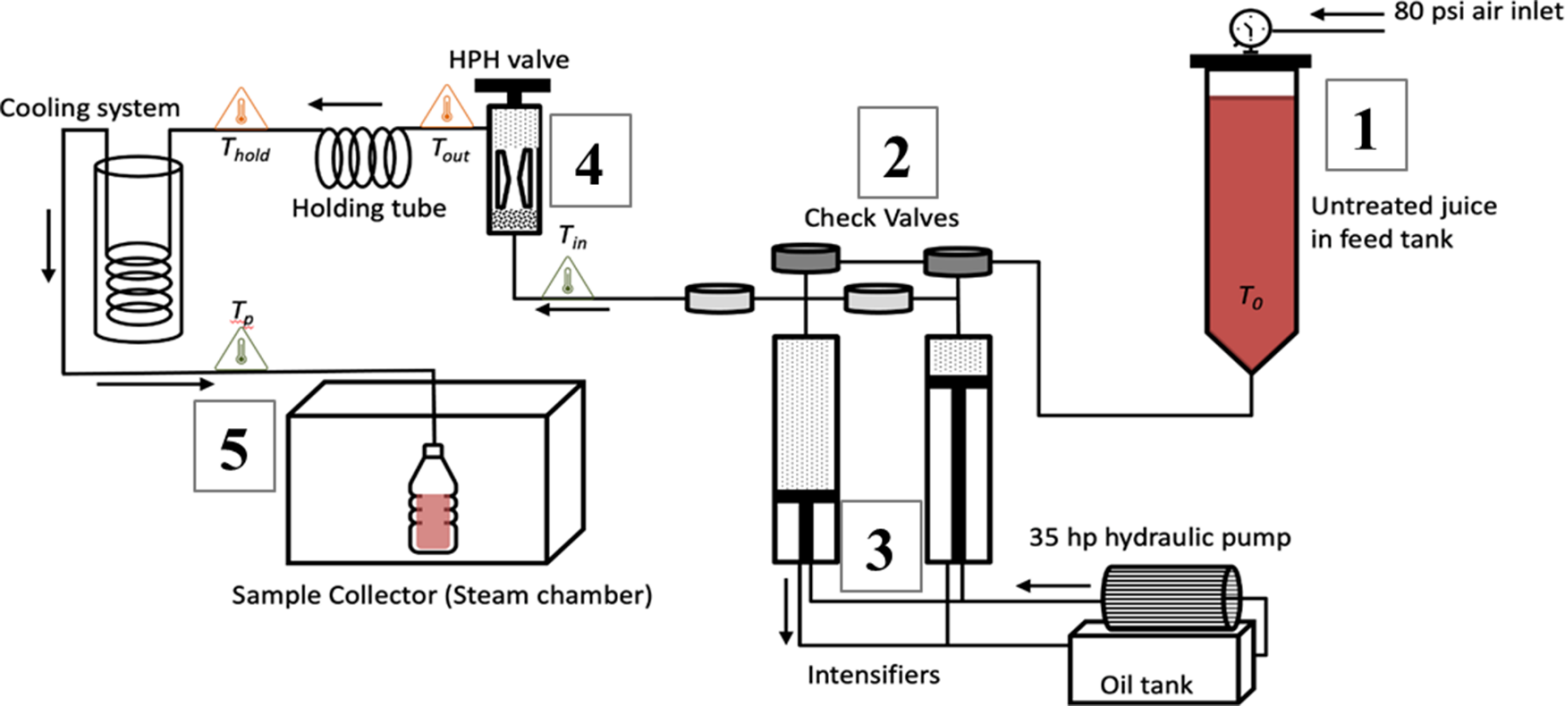
Experimental flow for continuous flow, high pressure homogenization. *T*_0_ = Inlet juice temperature. *T*_in_ = Juice temperature (pressurized juice inside the system) before entering the throttling valve. *T*_out_ = Juice temperature after exiting the throttling valve (temperature rise because of shear, pressure drop, and turbulence). *T*_hold_ = Juice temperature after holding. *T*_p_ = Juice temperature after cooling. HPH = High pressure homogenization. (1) Product inlets/feed tanks: The intensifiers are filled with the product from the inlets/feed tanks, which requires some pressure differential. An 80 psi air inlet compressor is used to release the sample to the intensifiers. (2) Check valves: To prevent backflow either from the intensifiers to the product inlet or between different intensifier chambers, check valves are used. The system has 4 active check valves that only allow flow when engaged. (3) Intensifier array: The intensifier array for this experiment comprises two single-acting intensifiers that are assisted with a 35 hp hydraulic pump to increase the pressure. By coupling them with active valves, a 3-part cycle (intake, compression, and exhaust) is created in each cylinder. (4) Pressure release component: The pressure release component is designed to restrict the flow of the blueberry juice and can take multiple forms. The fluid enters an area of greater volume, resulting in cavitation, which is one of the principal mechanisms involved in the changes induced by CFHPH. The system has a homogenizing valve. (5) Sample cooling and collection: At the end of the process, the samples go through a cooling system and are collected in a steam chamber to ensure that there is no outer influence on the sample.

Blueberry juice was treated in a pilot-scale dual-intensifier CFHPH unit (model nG7900, Stansted Fluid Power Ltd., Harlow, Essex, UK) equipped with a micrometering needle valve (model 60VRMM4882, Autoclave Engineers, Fluid Components, Erie, PA, USA), which consists of a hydraulic feed pump to maintain constant pressure to the intensifiers, which alternately takes in fluid product and discharges the pressurized fluid to the desired pressure (200, 250, and 300 MPa) with an inlet temperature (4 or 22 °C). Pressurized fluid was forced through the narrow orifice of the throttling valve that was adjusted to achieve desired flow rates (0.75, 1.125, and 1.5 L/min). The parameters (pressure level, flow rate, and inlet temperature) for CFHPH were chosen based on our previous research,^30^ which provided the intended residence time for pasteurization. With immediate pressure drop after valve fluid temperature rises resulting in exit temperature ranging from 50 ± 2 to 84 ± 2 °C (Table 1), which then entered a hold tube with a residence time of 10–20 s, following immediate cooling by immersing tubular heat exchanger in an ice–water bath to final product temperature of 10 °C and collected in sterile 100 mL bottles inside steam chamber (Figure 1).

**Table 1:** Experimental Design and Processing Parameters^a^

treatment				
CFHPH				
*T*_0_ (°C)	pressure (Mpa)	flow rate (L/min)	*T*_1_ (°C)	*T*_2_ (°C)
4	200	0.75	10.00 ± 2.00	50.00 ± 2.00
		1.125	10.00 ± 2.00	52.00 ± 2.00
		1.50	10.00 ± 2.00	54.00 ± 2.00
	250	0.75	10.00 ± 2.00	60.00 ± 2.00
		1.125	10.00 ± 2.00	62.00 ± 2.00
		1.50	10.00 ± 2.00	64.00 ± 2.00
	300	0.75	10.00 ± 2.00	70.00 ± 2.00
		1.125	10.00 ± 2.00	72.00 ± 2.00
		1.50	10.00 ± 2.00	74.00 ± 2.00
22	200	0.75	22.00 ± 2.00	60.00 ± 2.00
		1.125	22.00 ± 2.00	62.00 ± 2.00
		1.50	22.00 ± 2.00	64.00 ± 2.00
	250	0.75	22.00 ± 2.00	70.00 ± 2.00
		1.125	22.00 ± 2.00	72.00 ± 2.00
		1.50	22.00 ± 2.00	74.00 ± 2.00
	300	0.75	22.00 ± 2.00	80.00 ± 2.00
		1.125	22.00 ± 2.00	82.00 ± 2.00
		1.50	22.00 ± 2.00	84.00 ± 2.00

HTST		
	*T* (°C)	time (s)
	75	15
	85	
	95	

a CFHPH = continuous flow high pressure homogenization; HTST = high temperature short time pasteurization; *T* = juice temperature in the HTST system; *T*_0_ = juice temperature when entering the system; *T*_1_ = juice temperature (pressurized juice inside the system) before entering the throttling valve; *T*_2_ = juice temperature after exiting the throttling valve (temperature rise because of shear, pressure drop, and turbulence).

#### 2.2.3 High Temperature Short Time Processing

**Figure 2:**
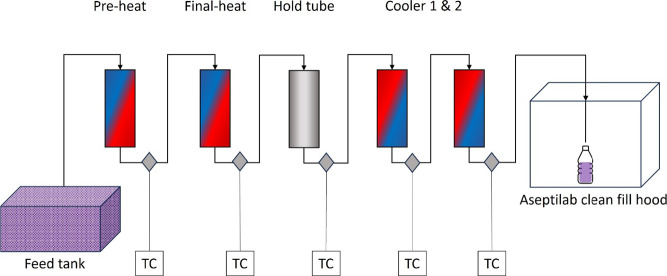
Schematic diagram of the HTST unit. Final heat = 75 °C, 85 °C, and 95 °C. Hold time = 15 s.

HTST processing was carried out using a pilot-scale pasteurization system (MicroThermics Model: UHT/HTST-DH, Raleigh, NC, USA), which electrically heated to varied temperatures (75, 85, and 95 °C) with a 0.5 L/min flow rate for 15 s residence time followed by immediate cooling to 10 °C and collected in 100 mL sterile bottles inside Aseptic lab Clean Fill Hood (Figure 2, Table 1). In line with industry standards,^31^ and FDA recommendations for juice pasteurization (FDA, 2001) to effectively inactivate microorganisms while preserving fruit juices’ sensory and nutritional qualities, and previous studies,^32,33^ we selected HTST treatment parameters that also closely matched the CFHPH treatment combinations applied in this research.

#### 2.2.4 Juice Sampling and Storage

The control (untreated), thermally (HTST), and pressure (CFHPH)-treated blueberry juices were collected in 100 mL of sterile Corning HDPE Round polypropylene bottles (Corning, Inc., Corning, NY, USA) and stored at market-simulated refrigerated conditions at 4 °C for 45 days of storage. Samples were withdrawn every 15 days and stored at −80 °C until further analysis.

### 2.3 Phytochemical Analysis

#### 2.3.1 Anthocyanin Analysis Using UHPLC-DAD-MS

Blueberry juice samples (5 mL) were extracted with 5 mL of mixed solvent containing methanol: water: formic acid at 50:48:2 (v/v/v), respectively, for anthocyanin analysis. Extraction was conducted under dark conditions at room temperature. Samples were homogenized for 2 min at 4480 × *g*, sonicated in ice-cold water for 30 min, and centrifuged for 10 min at 7826 × *g*. The resulting supernatants were pooled and filtered through a 0.45-μm polytetrafluoroethylene membrane. The final volume was measured, and the sample was kept at −80 °C for further analysis.

Anthocyanins were analyzed with the 1290 Agilent Infinity II ultrahigh performance liquid chromatography system coupled to a diode array detector (UHPLC-DAD) (Agilent, Santa Clara, CA, USA). The separation of target compounds was carried out on a Zorbax RRHD Extend-C18 column (2.1 × 100 mm, 1.8 μm) at 25 °C with a flow rate of 0.3 mL/min using a binary mobile phase consisting of (A) 0.2% trifluoroacetic acid (TFA) in water and (B) 50% acetonitrile and 0.2% TFA in water. The following gradient program was used to achieve the separation of anthocyanin: 20% B (0–1 min), 20–32% B (1–2 min), 32–38% B (2–4 min), 38–50% B (4–6 min), 50–60% B (6–7 min), 60–80% B (7–8 min), 80–20% B (8–9 min), and 20% B (9–10 min). The optical densities of the compounds were monitored at 520 nm. Delphinidin 3-glucoside, malvidin 3-glucoside, petunidin 3-glucoside, peonidin 3-glucoside, and cyanidin 3-glucoside standards were used for calibration and quantitation of all identified anthocyanins in UHPLC. Each anthocyanin was expressed as equivalent to the same anthocyanidins in mg per 100 mL of blueberry juice. The total anthocyanin was measured by adding all individual anthocyanin contents.

Mass spectral (MS) analysis was performed on a maXis impact mass spectrometer (Bruker Daltonics, Billerica, MA, USA) using electrospray ionization in positive ionization mode according to Singh et al.^34^ MS and broadband collision-induced dissociation (bbCID) data were acquired in the *m*/*z* 50–2000 scan range. The capillary voltage of the ion source was 3.5 kV, the drying gas flow rate was 8.0 L/min, and the nebulizer gas pressure was 0.32 MPa. Nitrogen was used as a nebulizing and drying gas. The accurate mass data for the precursor ions were processed using Data Analysis 4.3 software (Bruker Daltonics, Billerica, MA, USA).

#### 2.3.2 Ascorbic Acid Analysis

Ascorbic acid extraction was performed according to Chebrolu et al.^35^ Briefly, blueberry juice (5 mL) was extracted with 5 mL of 3% metaphosphoric acid. Samples were homogenized for 2 min at 4480 × *g*, sonicated in ice-cold water for 30 min, and centrifuged for 10 min at 7826 × *g*. The supernatant was poured into a 50 mL tube, and the final volume was recorded. The extracted sample was filtered through 0.45 μm polytetrafluoroethylene membrane, and 600 μL was transferred into amber-colored HPLC vials for ascorbic acid analysis. Another 300 μL of the sample was mixed with 300 μL of tris(2-carboxyethyl) phosphine solution for dehydroascorbic acid (DHA) analysis.

High-performance liquid chromatography with photodiode array (HPLC-PDA) analysis was carried out using an Agilent 1220 series HPLC system with an Eclipse Plus C18 column (250 × 4.6 mm, 5 μm), and a PDA detector was used with isocratic elution using 0.3 M aqueous phosphoric acid as a mobile phase A. With a flow rate of 0.4 mL/min, a 10 μL sample was injected into the column. The peaks were monitored at 210 and 243 nm with a run time of 18 min. Results were expressed as milligrams of ascorbic acid per 100 mL of blueberry juice.

### 2.4 Polyphenol Oxidase Activity

Exactly 5 mL of blueberry juice was extracted with 5 mL of 0.05 M potassium phosphate buffer (pH 7.0). The mixture was centrifuged at 10,000 × *g* for 10 min at 4 °C. The supernatant was collected and filtered through a 0.45 μm syringe filter (Chromafil Xtra PTFE-45/25, Macherey-Nagel GmbH & Co. KG, Düren, Germany), and 5 mL of chilled acetone: water (1:1, v/v) was added for enzyme extraction. The solution was again centrifuged at 10,000 × *g* for 10 min at 4 °C. Then, 2 mL of 0.05 M potassium phosphate buffer (pH 7.0) was added to the precipitated residue enzymes. For 96-well plates, 150 μL of phosphate buffer, 25 μL of enzyme extract, and 25 μL of substrate [substrate, 0.1 M pyrocatechol] was added, and change in absorbance at 405 nm for 5 min [1 unit = 0.001 change in absorbance/min] was recorded using a UV–vis spectrophotometer.

### 2.5 Statistical Analysis

One-way analysis of variance (ANOVA) with generalized linear models was performed on all the data sets. Each statistical analysis involved triplicate samples processed as independent experiments, each with two replicates analyzed for quality. Tukey’s Honest Significant Difference (HSD) test was applied at a 5% significance level (α = 0.05) to determine treatment and storage differences. Principal component analysis (PCA) was conducted to assess the impact of CFHPH and HTST processing on the levels of anthocyanins, ascorbic acid, and PPO activity in blueberry juice. All statistical analyses were performed using XLSTAT software, version 2022 (Addinsoft, New York, NY, USA).

## 3 Results and Discussion

### 3.1 Impact of Processing on Ascorbic Acid Stability

We tested three different HTST conditions, 75, 85, and 95 °C, with untreated samples. On day 0 of storage, HTST blueberry juice had significantly (*P* ≤ 0.05) less ascorbic acid content than the untreated sample; for example, the juice treated at 75 °C had 57% less ascorbic acid than the untreated control (3.71 mg/100 mL compared with 8.64 mg/100 mL) (Table 2). This aligns with previous findings that high temperature causes degradation of ascorbic acid in thermally processed juice.^8,36^

**Table 2:** Least Square means of the Ascorbic Acid Content in the Blueberry Samples after Processing for each Storage Period^a^

CFHPH			total ascorbic acid content (mg/100 mL)				
*T*_0_ (°C)	pressure (MPa)	flow rate (L/min)	storage at 4 °C (days)				Pr > F (model)
			0	15	30	45	
4	200	0.75	6.11^cdef-A^	4.45^cde-A^	4.4^abcde-AB^	1.29^fghi-B^	0.001
		1.125	6.04^cdefg-A^	3.55^cde-B^	2.93^cde-BC^	1.81^efghi-C^	<0.0001
		1.5	5.10^defgh-A^	4.47^cde-A^	4.73^abcde-A^	1.38^fghi-B^	<0.0001
	250	0.75	7.95^abc-A^	4.97^bcde-A^	4.71^abcde-A^	1.06^hi-B^	0.000
		1.125	6.09^cdefg-A^	3.17^de-B^	3.44^cde-B^	0.67^i-C^	<0.0001
		1.5	4.54^fgh-A^	5.03^bcde-A^	2.55^de-B^	0.76^i-C^	<0.0001
	300	0.75	6.97^bcde-A^	4.43^cde-B^	3.62^bcde-B^	1.13^ghi-C^	<0.0001
		1.125	4.85^efgh-A^	3.88^cde-A^	4.13^abcde-A^	1.18^fghi-B^	0.000
		1.5	7.55^abc-A^	4.77^bcde-AB^	3.6^bcde-B^	2.76^defg-B^	0.003
22	200	0.75	9.16^a-A^	7.39^ab-AB^	5.67^abc-B^	5.28^ab-B^	0.001
		1.125	6.92^bcde-A^	6.33^abc-B^	6.54^a-B^	5.52^ab-B^	0.004
		1.5	6.91^bcde-A^	6.08^abc-AB^	5.04^abcd-BC^	4.67^bc-C^	<0.0001
	250	0.75	8.49^ab-A^	5.52^abcd-B^	5.32^abcd-B^	2.59^defgh-C^	<0.0001
		1.125	7.71^abc-A^	6.23^abc-B^	6.25^ab-B^	6.36^a-B^	0.000
		1.5	7.4^abc-A^	3.46^cde-B^	4.58^abcde-B^	4.57^bc-B^	<0.0001
	300	0.75	8.12^abc-A^	6.09^abc-BC^	5.6^abcd-BC^	4.13^bcd-C^	<0.0001
		1.125	7.23^abcd-A^	5.23^abcd-B^	5.59^abcd-B^	5.48^ab-B^	0.005
		1.5	7.88^abc-A^	8.04^a-A^	4.99^abcde-B^	3.42^cde-B^	<0.0001
HTST	75 °C	15 s	3.71^h-A^	2.3^e-B^	2.74^de-AB^	2.21^efgh-B^	0.009
	85 °C		3.95^gh-A^	2.81^de-B^	2.10^e-BC^	1.84^efghi-C^	0.000
	95 °C		4.09^fgh-A^	3.09^de-AB^	3.06^cde-AB^	2.82^def-B^	0.003
control			8.64^ab-A^	6.21^abc-B^	5.44^abcd-B^	5.77^ab-B^	0.004
Pr > F (model)			<0.0001	<0.0001	<0.0001	<0.0001	

a CFHPH = continuous flow high pressure homogenization; HTST = high temperature short time pasteurization. Different capital letters signify significant differences across storage days, while lowercase letters indicate significant differences among treatments on that specific storage day.

Although the HTST juices had lower levels of ascorbic acid than the controls, the residual ascorbic acid was relatively stable, and its stability was higher in samples treated at higher temperatures within the HTST process (Table 2). For example, after 45 days of storage, HTST juice treated at 95 °C retained 32.6% of the ascorbic acid present in untreated samples at day 0, but HTST juice treated at 75 °C retained only 25.57% of the ascorbic acid. The higher stability of ascorbic acid in blueberry juice processed at 95 °C might be due to thermal inactivation of ascorbic acid degradation enzymes, such as ascorbic acid oxidase (AAO) and dehydroascorbate reductase (DHR).^37^ Indeed, blanching of fruits and vegetables at around 85–95 °C is known to inactivate AAO.^38^ In addition, ascorbic acid is more stable at lower pH, and HTST may influence the pH of the juice, thus enhancing ascorbic acid stability.^39^ Ascorbic acid levels varied in CFHPH-treated samples, indicating the influence of the inlet temperature. For CFHPH, we tested a range of pressures, inlet temperatures, and flow rates (Table 1, Figure 1). The levels of ascorbic acid were higher in samples treated with an inlet temperature of 22 °C compared with 4 °C (Table 2). For instance, increasing the inlet temperature from 4 to 22 °C at 300 MPa and a flow rate of 1.125 L/min resulted in a statistically significant (*P* ≤ 0.05) rise in total ascorbic acid from 4.85 to 7.23 mg/100 mL (Table 2). This may be due to the limited oxidation of ascorbic acid, as an increase in temperature leads to a decline in the solubility of oxygen.^40^ Indeed, the oxygen solubility in water at 4 and 22 °C is 10.2 and 7.6 mg/L, respectively.^41^ The effect of the flow rate on the ascorbic acid content was found to be variable. Increasing the flow rate from 0.75 to 1.5 L/min at 200 MPa and an inlet temperature of 22 °C resulted in a significant rise in total ascorbic content from 6.91 to 9.16 mg/100 mL. However, pressure levels at 250 and 300 MPa did not have a significant impact on ascorbic acid content by flow rate at an inlet temperature of 22 °C. Inlet temperature was found to have a major impact on the stability of ascorbic acid. CFHPH treatment at 22 °C inlet temperature retained higher ascorbic acid content than CFHPH treatment at inlet temperature of 4 °C across all pressure and flow rate levels as well as HTST samples.^20^ Overall, CFHPH (200 MPa, 1.25 L/min, 22 °C) resulted in a 26.3% reduction in total vitamin C content after 45 days, exceeding the 31.05% decrease in orange juice observed with pulsed electric field (PEF) processing at 35 kV/cm/750 μs after 40 days of cold storage.^42^

### 3.2 Impact of Processing on Anthocyanin Stability

**Figure 3:**
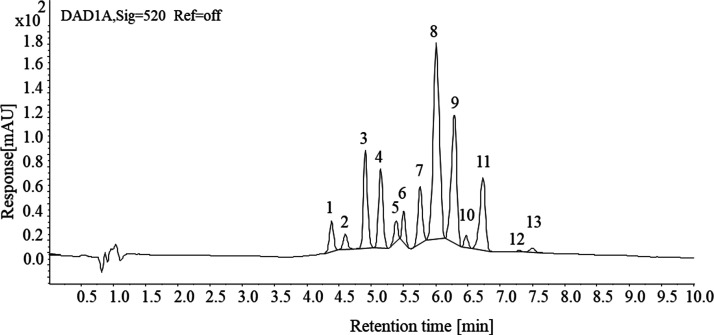
Ultra high-performance liquid chromatogram of 13 identified anthocyanins in blueberry juice. Peak 1, delphinidin 3-galactoside; Peak 2, delphinidin 3-glucoside; Peak 3, cyanidin 3-galactoside; Peak 4, delphinidin 3-arabinoside; Peak 5, petunidin 3-galactoside; Peak 6, cyanidin 3-arabinoside; Peak 7, peonidin 3-galactoside; Peak 8, peonidin-3-glucoside; Peak 9, malvidin-3-galactoside; Peak 10, malvidin-3-glucoside; Peak 11, malvidin-3-arabinoside; Peak 12, peonidin-3-(6″-acetyl)glucoside; and Peak 13, delphidinin-3-(6″-malonyl)glucoside.

Blueberry juice contains 13 types of anthocyanin, including anthocyanidin glycosides of cyanidin, delphinidin, malvidin, peonidin, petunidin, and acylated anthocyanins. Comparing mass spectra, elution order, and absorbance spectra of each anthocyanin molecule using UPLC-ESI-MS, these anthocyanins were identified in blueberry juice in the order of elution (Figure 3) as delphinidin 3-galactoside, delphinidin 3-glucoside, cyanidin 3-galactoside, delphinidin 3-arabinoside, petunidin 3-galactoside, cyanidin 3-arabinoside, peonidin 3-galactoside, peonidin-3-glucoside, malvidin-3-galactoside, malvidin-3-glucoside, malvidin-3-arabinoside, peonidin-3-(6″-acetyl) glucoside, and delphidinin-3-(6″-malonyl) glucoside. Peonidin and malvidin made up more than 35 and 25% of total anthocyanin, respectively; delphinidin and cyanidin made up less than 12% of total anthocyanin.

At day 0 of storage, the total anthocyanin contents in control (untreated), pasteurized (HTST), and pressure-treated (CFHPH) blueberry juice ranged from 64.11 to 148.03 mg/100 mL, with the highest amount of anthocyanins observed in blueberry juice treated with HTST at 95 °C for 15 s, while untreated samples (control) had the lowest amount. Within the HTST samples, the total anthocyanin content was significantly higher as the treatment temperature increased from 75 °C (86.82 mg/100 mL) to 95 °C (148.03 mg/100 mL; Table 3). Anthocyanins are thermostable at 80–120 °C for a limited time at low pH.^43^ The initial increase in anthocyanin content may be attributed to the HTST treatment causing a reduction in viscosity of the liquid phase, thermal expansion of the cell, thermal breakdown of the cell wall, and the release of bound anthocyanins from cellular structures, and improved activities of cell-wall hydrolyzing enzymes, which together facilitate anthocyanin release.^44−46^ The findings are consistent with another nonthermal processing method known as thermosonication, which resulted in 94.12% increase in anthocyanin levels compared to control samples.^47^

**Table 3:** Least Square Means of Total Anthocyanin Content in the Blueberry Samples after Processing for each Storage Period^a^

CFHPH			total anthocyanin content (mg/100 mL)				
*T*_0_ (°C)	pressure (MPa)	flow rate (L/min)	storage at 4 °C (days)				Pr > F (model)
			0	15	30	45	
4	200	0.75	94.38^bcdef-A^	72.72^bcdefgh-B^	70.82^bcdef-B^	73.20^bcde-B^	<0.0001
		1.125	88.08^cdefg-A^	70.61^efgh-AB^	55.56^defg-BC^	45.95^ghij-C^	<0.0001
		1.5	93.79^bcdef-A^	79.07^bcdefgh-A^	52.15^defg-B^	43.13^hij-B^	<0.0001
	250	0.75	103.87^bcd-A^	89.07^abcde-B^	80.32^abcd-B^	58.36^defghi-C^	<0.0001
		1.125	100.61^bcde-A^	86.04^bcdef-B^	83.99^abc-B^	59.13^defghi-C^	<0.0001
		1.5	104.97^bcd-A^	84.99^bcdef-B^	84.53^abc-B^	62.19^cdefgh-C^	<0.0001
	300	0.75	113.07^b-A^	98.54^abc-B^	99.90^a-B^	70.76^bcde-C^	<0.0001
		1.125	110.96^bc-A^	101.09^ab-B^	94.39^ab-B^	65.45^cdefg-C^	<0.0001
		1.5	111.39^bc-A^	96.9^abcd-B^	98.79^a-B^	93.60^ab-B^	<0.0001
22	200	0.75	79.02^efg-A^	58.29^ghi-B^	48.42^fg-BC^	39.04^ij-C^	<0.0001
		1.125	70.69^fg-A^	56.85^hi-AB^	49.47^efg-AB^	45.98^ghij-B^	0.02
		1.5	90.47^bcdef-A^	70.36^efgh-AB^	63.68^cdef-B^	52.47^fghi-B^	0.002
	250	0.75	73.02^fg^	73.981^cdefgh^	62.16^cdef^	54.60^efghi^	0.057
		1.125	86.07^defg-A^	72.62^defgh-A^	66.62^cdef-A^	40.46^ij-B^	<0.0001
		1.5	91.34^bcdef-A^	92.79^abcde-A^	81.87^abc-A^	48.07^ij-B^	<0.0001
	300	0.75	87.67^cdefg^	61.19^fghi^	73.87^bcde^	66.13^cdefg^	0.143
		1.125	84.97^defg-A^	77.29^bcdefgh-AB^	67.33^cdef-AB^	53.18^efghi-B^	0.038
		1.5	104.94^bcd-AB^	111.45^a-A^	86.39^abc-BC^	77.934^bcd-C^	0.001
HTST	75 °C	15 s	86.82^cdefg^	82.07^bcdefg^	78.61^abcd^	72.85^cdef^	0.556
	85 °C		100.48^bcde-A^	86.42^bcde-B^	84.66^abc-B^	82.42^abc-B^	0.003
	95 °C		148.03^a-A^	86.38^bcde-B^	81.34^abcd-B^	81.24^abc-B^	<0.0001
control			64.11^g-A^	37.92^i-B^	34.99^g-B^	26.88^j-B^	<0.0001
Pr > F (model)			<0.0001	<0.0001	<0.0001	<0.0001	

a CFHPH = continuous flow high pressure homogenization; HTST = high temperature short time pasteurization. Different capital letters signify significant differences across storage days, while lowercase letters indicate significant differences among treatments on that specific storage day.

We further examined anthocyanins over time in the different samples, which were stored at 4 °C. After 15 days of cold storage, blueberry juice treated at 95 °C showed a statistically significant degradation (*P* ≤ 0.05) of anthocyanins, decreasing from 148.03 mg/100 mL at day 0 to 86.38 mg/100 mL at day 15. After this initial storage period, the HTST treatment temperature had a statistically insignificant effect on the anthocyanin degradation. This finding suggests that while high temperatures initially led to high anthocyanin contents, the subsequent degradation offset this effect.^43^ This can be mainly attributed to the nonenzymatic conversion of monomeric anthocyanins into more condensed compounds during storage, where anthocyanins covalently associate with other flavanols or organic acids, leading to the formation of a new pyran ring by cycloaddition.^48^ Indeed, previous research found that during cold storage, temperature-induced degradation of anthocyanins can result from oxidation, structural changes, and the formation of condensation products.^49^

The anthocyanin content and stability in the CFHPH samples varied depending on the pressure level, flow rate, and inlet temperature. Pressure levels above 250 MPa enhanced the anthocyanin content, highlighting the critical role of pressure in retaining these compounds. Specifically, at an inlet temperature of 4 °C and a flow rate of 1.5 L/min, the initial total anthocyanin content increased from 93.79 to 111.39 mg/100 mL when the pressure was increased from 200 to 300 MPa. After 45 days of storage, the total anthocyanin content significantly decreased to 43.13 mg/100 mL in samples treated with 200 MPa pressure, whereas it was maintained at 93.6 mg/100 mL in samples treated with 300 MPa pressure. Blueberry juice treated at 300 MPa, with a flow rate of 1.5 L/min and an inlet temperature of 4 °C, had the highest stability of individual anthocyanins during storage (Figure S1). The positive effect of pressure homogenization on anthocyanin stability aligns with previous research showing that high pressure can release bound anthocyanins from cellular structures, making them more accessible.^50^

In the CFHPH system, the effect of the flow rate on anthocyanin content and stability varied depending on the pressure and inlet temperature. For instance, increasing the flow rate from 0.75 to 1.5 L/min at 300 MPa and inlet temperature of 22 °C resulted in a rise in total anthocyanin from 87.67 to 104.94 mg/100 mL. This increase may be due to improved mass transfer and the rise in temperature associated with higher flow rates, allowing for the release of more anthocyanins.^51^ Additionally, after 45 days of storage, at a flow rate of 1.5 L/min, the total anthocyanin content decreased to 77.93 mg/100 mL, whereas it decreased to 66.13 mg/100 mL with a flow rate of 0.75 L/min after 45 days of storage. However, when the flow rate was increased at an inlet temperature of 4 °C, the concentration and stability of anthocyanin fluctuated and were influenced by the combined impact of processing and the initial concentration of ascorbic acid.

Likewise, the inlet temperature played an important role in anthocyanin content and stability in the CFHPH system. On day 0 of storage, individual anthocyanin and total anthocyanin concentrations were higher when blueberry juice was treated at 300 MPa with an inlet temperature of 4 °C (Table 3, Figure S1). An inlet temperature of 22 °C resulted in lower anthocyanin levels compared to samples with a 4 °C inlet temperature for all pressure and flow rate levels. For instance, increasing the inlet temperature from 4 to 22 °C at 250 MPa and a flow rate of 1.125 L/min resulted in a decrease in total anthocyanin from 100.61 to 86.07 mg/100 mL (Table 2). Also, the storage stability improved with the inlet temperature at 4 °C, where it only slightly decreased to 59.13 mg/100 mL, whereas it decreased to 40.46 mg/100 mL with the inlet temperature at 22 °C after 45 days of cold storage. As mentioned earlier, when the inlet temperature was 4 °C, the degradation of ascorbic acid in blueberry juice accelerated, and this was associated with an increment in anthocyanin content.^52^ This is because ascorbic acids are electrophilic compounds and are speculated to attack the same nucleophilic sites of anthocyanin.^53^

Overall, the untreated juice showed the lowest anthocyanin stability compared to the HTST and CFHPH treatment at 300 MPa. Buckow et al.^10^ also showed that untreated juice has an almost 10-fold higher anthocyanin degradation rate compared to pasteurized juice when stored at 4 °C. This may be because high pressure increases the mass transfer rate by increasing cell permeability and diffusion of secondary metabolites, leading to higher extraction of anthocyanins. The same principle applies to HTST treatment, which increases anthocyanin extraction.^54^ These findings demonstrate that anthocyanin levels increase with high pressure in the CFHPH system, making it a promising method for blueberry juice production. Overall, CFHPH (300 MPa, 1.5 L/min, 4 °C) treatment resulted in higher anthocyanin retention compared to that of the HTST and untreated sample after 45 days of cold storage. The different effects of these processing methods can be attributed to their mechanisms. HTST treatment relies primarily on temperature, while CFHPH combines pressure, flow rate, and temperature. Also, removing oxygen from the juice could prevent anthocyanin degradation during storage, as shown in blueberry puree processed under oxygen-free conditions, highlighting the need for further optimization efforts.^36^

**Figure 4:**
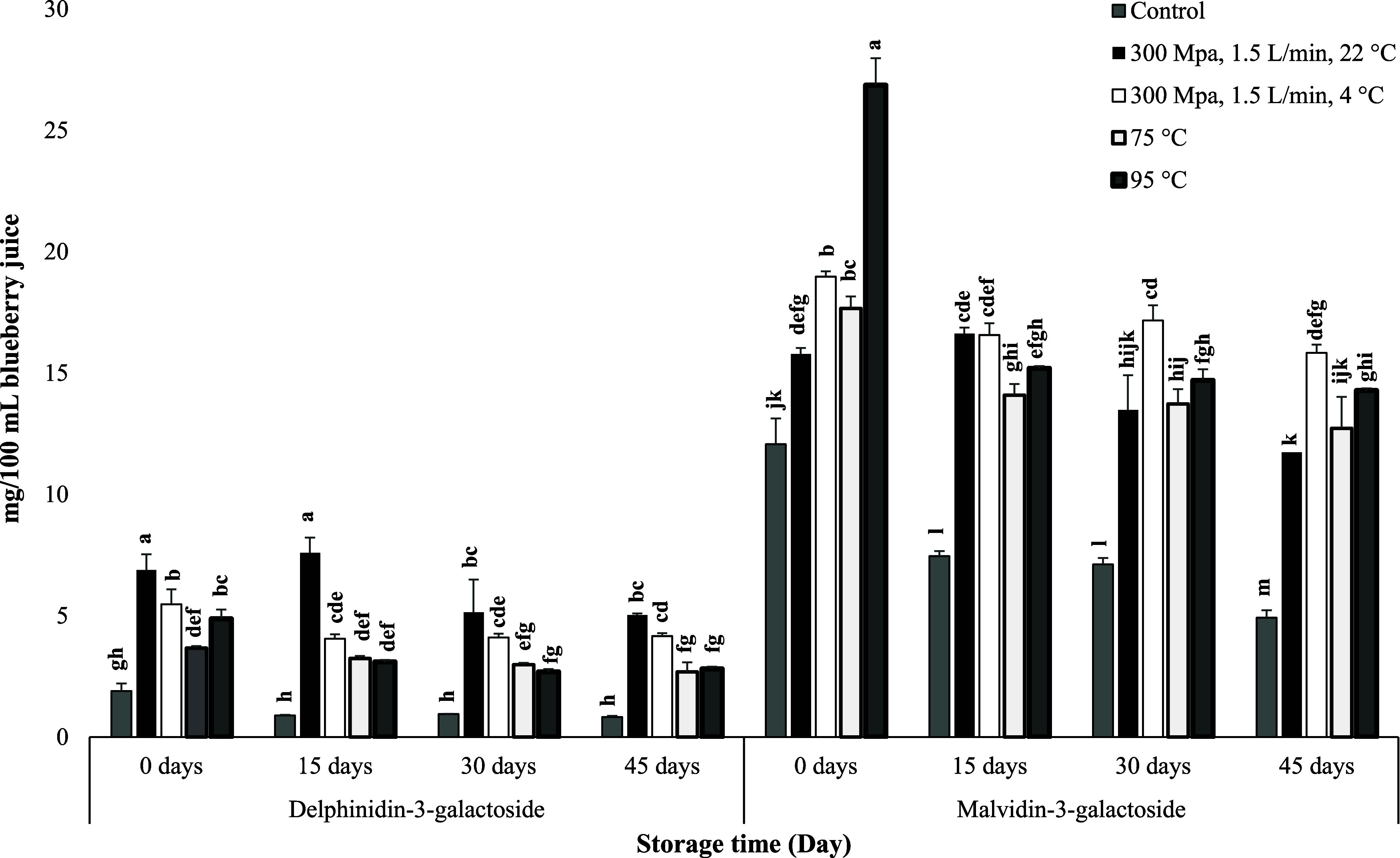
Bar graph for least-squares means of delphinidin 3-galactoside and malvidin 3-galactoside content in blueberry juice after processing and storage at 4 °C for 45 days. Different lowercase letters signify significant differences among treatments during storage.

We also observed that individual anthocyanins responded differently to changing CFHPH treatment variables, with the impact of processing varying based on the number of hydroxyl (−OH) and methoxy (−OCH_3_) groups present in the anthocyanins. Hydroxyl groups were found to be more susceptible than methoxy groups at higher temperatures.^55^ For example, the processing had a stronger effect on delphinidin than malvidin, likely because delphinidin has two hydroxyl groups, while malvidin has two methoxy groups at positions 3′ and 5′ of the B-ring. Specifically, CFHPH at 300 MPa pressure with an inlet temperature of 22 °C and a flow rate of 1.5 L/min, delphinidin 3-galactoside significantly increased by 2.63-fold, while malvidin 3-galactoside only increased by 0.31-fold (Figure 4).

Moreover, we found that increasing the inlet temperature in the CFHPH system decreased malvidin 3-galactoside and increased delphinidin 3-galactoside at 300 MPa pressure and 1.5 L/min flow rate, indicating that the synergistic effect of pressure and temperature favored delphinidin content in the CFHPH system.

### 3.3 Impact on PPO Activity

**Figure 5:**
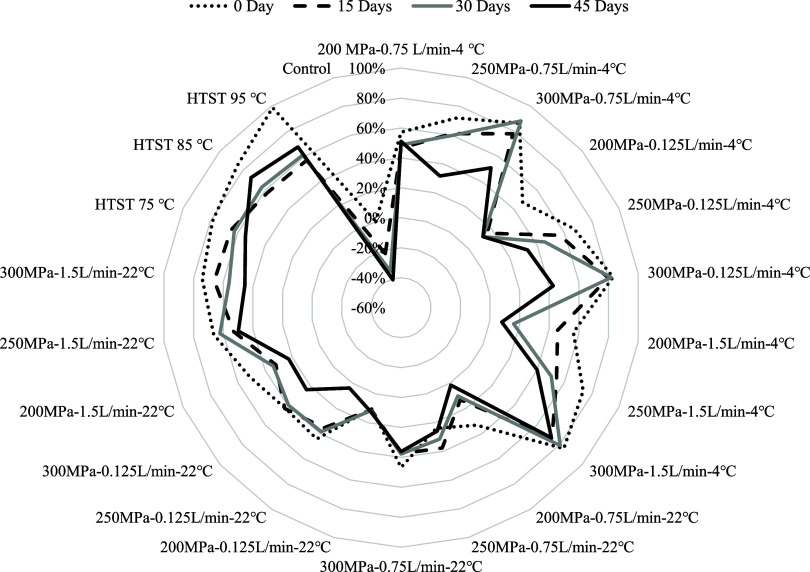
Radar plot for a percentage reduction in polyphenol oxidase (PPO) activity of blueberry juice after treatment and storage at 4 °C. HTST, high temperature short time pasteurization.

Polyphenol oxidase (PPO) plays a significant role in the degradation of anthocyanins in blueberry juice, as it catalyzes the oxidation of phenolic compounds, resulting in the formation of oxidative products such as 4-methylcatechol, which further accelerates anthocyanin degradation.^56^ HTST treatment at 95 °C resulted in the highest reduction of PPO activity by 98.75% (Figure 5).

The decrease in PPO activity through HTST treatment was positively correlated with the increasing temperature. The high-temperature treatment effectively inactivated the small amount of previously undetected, temperature-resistant oxidative enzymes present in the blueberry juice.^10^ Also, high temperatures cause thermal inhibition of enzyme activity, leading to inactivation through change in their 3D structure and a decrease in their ability to catalyze reactions.^57^

In our CFHPH system, we observed a substantial decrease in PPO activity (Figure 5). For example, blueberry juice samples treated at 300 MPa, across all flow rates and at an inlet temperature of 4 °C, showed a greater than 80% decrease in PPO activity, while CFHPH treatment at 200 MPa pressure, at various flow rates and inlet temperatures, had less than 57% reduction of PPO activity (Figure 5). Previous research on apple juice has shown similar results: as the HPH pressure increased from 200 to 300 MPa, the residual activity of enzyme decreased significantly by 70%.^58^ This reduction in PPO activity can be attributed to the alteration of the native PPO structure under high pressure conditions.^59,60^ However, the outcome opposes the effect of high hydrostatic pressure, which elevated PPO activity in blueberry puree within the range of 200–600 MPa.^61^ This might be due to the synergistic impact of the pressure and temperature during CFHPH processing. Therefore, the pressure and temperature maintained in the CFHPH positively impact anthocyanins in blueberry juice by effectively releasing bound anthocyanins and reducing PPO activity.^60,62^ Cold storage at 4 °C showed no detrimental effect but an increment in PPO activity with storage period, as shown in Figure 5. In particular, CFHPH at 300 MPa provides optimal conditions for the inactivation of PPOs, stability of anthocyanins, and subsequently better antioxidant activity.^63^ Overall, our results show that pressure treatment of 300 MPa at an inlet temperature of 4 °C across all flow rates successfully reduced PPO activity in blueberry juice at a level of inactivation comparable to that achieved by HTST treatment.^64^

### 3.4 Principal Component Analysis

**Figure 6:**
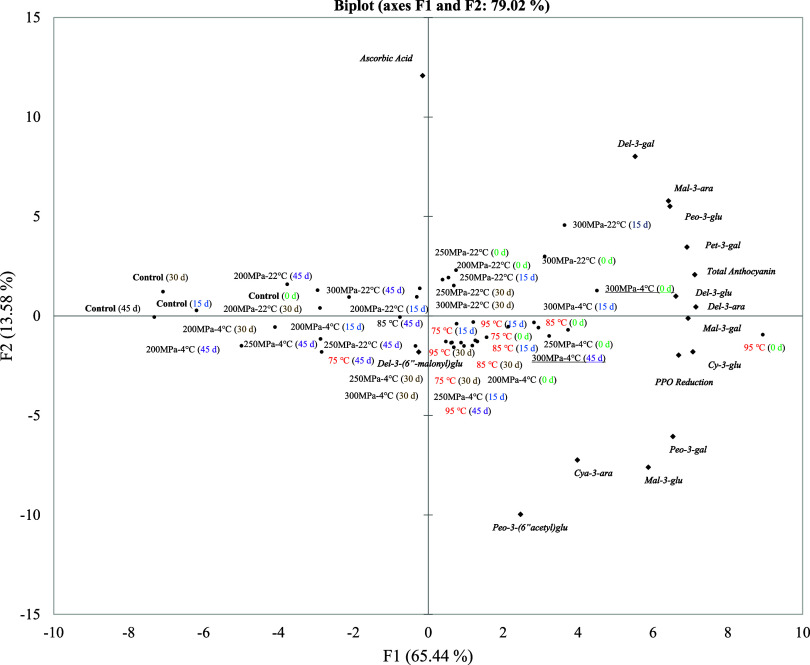
Principal component analysis (PCA) biplot showing average quality attribute values for blueberry juice samples treated with continuous flow high pressure homogenization (CFHPH) at 1.5 L/min flow rate and high temperature short time (HTST) treatments, followed by storage at 4 °C for 45 days. PPO, polyphenol oxidase; Del, delphinidin; Cya, cyanidin; Mal, malvidin; Peo, peonidin; Pet, petunidin; glu, glucoside; gal, galactoside; ara, arabinoside.

Principal component analysis (PCA) was conducted by using data from CFHPH treatment at a flow rate of 1.5 L/min, HTST, and control samples. Prior to PCA, the data underwent normalization through Min–Max scaling. A biplot (Figure 6) was generated to visualize the sample quality space. The first two principal components explained 79.02% of the total variation among the sample average scores. The samples were differentiated based on individual anthocyanins and total anthocyanins in component 1 and ascorbic acid in component 2. Overall, the sample quality space indicated that anthocyanins played a crucial role in indicating the sample variability for all storage periods. HTST treatment at 95 °C showed a higher amount of individual and total anthocyanins on day 0 compared to other treatments.

Moreover, at day 0, an increase in the pressure level from 200 to 300 MPa resulted in an increase in both individual and total anthocyanin content. Based on the impact on anthocyanin and its stability over 45 days of storage, CFHPH treatment at 300 MPa at 4 °C inlet temperature was the optimal condition for stabilizing juice quality during storage as compared to the traditional method of pasteurization. Overall, the PCA biplot suggests that CFHPH is the optimal method for stabilizing essential compounds like anthocyanin and ascorbic acid during cold storage which is not possible through thermal treatment.^45,63^

Therefore, the juice industry could greatly benefit from adopting CFHPH due to its ability to surpass HTST processing in preserving blueberry juice’s nutrients and health-promoting molecules (HPMs) during market-simulated storage. CFHPH’s preservation qualities meet the industry’s objective to provide nutrient-rich juices that meet the evolving preferences of consumers.^65^ Also, these emerging food processing technologies stand out in improving the sensory characteristics and consumer preference over traditional methods.^66^ As the market demands minimally processed, nutrient-dense options, blueberry juice stands out as a wholesome choice. However, to successfully implement CFHPH, it is crucial to conduct thorough research on optimal processing parameters and understand consumer preferences through sensory and consumer studies. By incorporating consumer feedback into decision-making processes, the industry can develop products that resonate with target markets, promoting competitiveness and profitability. Ultimately, the industry’s success in adopting CFHPH depends on its alignment with consumer preferences and industry needs.

In conclusion, the study reveals that CFHPH treatment of blueberry juice has temperature-dependent effects, favoring ascorbic acid retention at inlet temperature of 22 °C and anthocyanin retention at 4 °C. Pressure levels, particularly at 300 MPa, significantly impact the compound stability and enzyme activity. CFHPH effectively stabilizes nutrient compounds during storage, reduces PPO activity, and maintains nutritional integrity, making it a promising novel technology for enhancing fruit juice quality and nutritional value. Its continuous flow design and time efficiency make it a better option for processing juices consistently. Additionally, it is essential to minimize oxygen levels during juice processing and storage, as oxygen can negatively impact quality. Implementing techniques to remove oxygen in juice and system, such as inert gas flushing, can further enhance the quality and shelf life of the juice.

## Supporting Information Available

(XLSX)
Concentration of identified anthocyanins in various treated blueberry juices stored for 0, 15, 30, and 45 days at 4 °C (PDF)
