# Supplementary material for: Continuous-Flow High-Pressure Homogenization of Blueberry Juice Enhances Anthocyanin and Ascorbic Acid Stability during Cold Storage
Source: J Agric Food Chem. 2024 May 13;72(20):11629–39. doi: 10.1021/acs.jafc.4c01289 (PMC11117402; doi:10.1021/acs.jafc.4c01289)
Supplement: Supplementary file 2 — jf4c01289_si_002.pdf [file jf4c01289_si_002.pdf]

Supplement Information

Continuous-flow high-pressure homogenization of blueberry juice enhances anthocyanin and ascorbic acid stability during cold storage

Jayashan Adhikari[a], Lida Rahimi Araghi[b], Rakesh Singh[b], Koushik Adhikari[c], Bhimanagouda S. Patil[a*]

[a]*Vegetable and Fruit Improvement Center, Department of Horticultural Sciences, Texas A&M University, 1500 Research Parkway, Suite A120, College Station, TX 77845-2119, U.S.A.*

[b]*Department of Food Science and Technology, University of Georgia, 100 Cedar St., Athens, GA 30602, U.S.A.*

[c]*Department of Food Science and Technology, University of Georgia, 1109 Experiment St., Griffin, GA 30223, U.S.A.*

Corresponding Author: Bhimanagouda.Patil@ag.tamu.edu

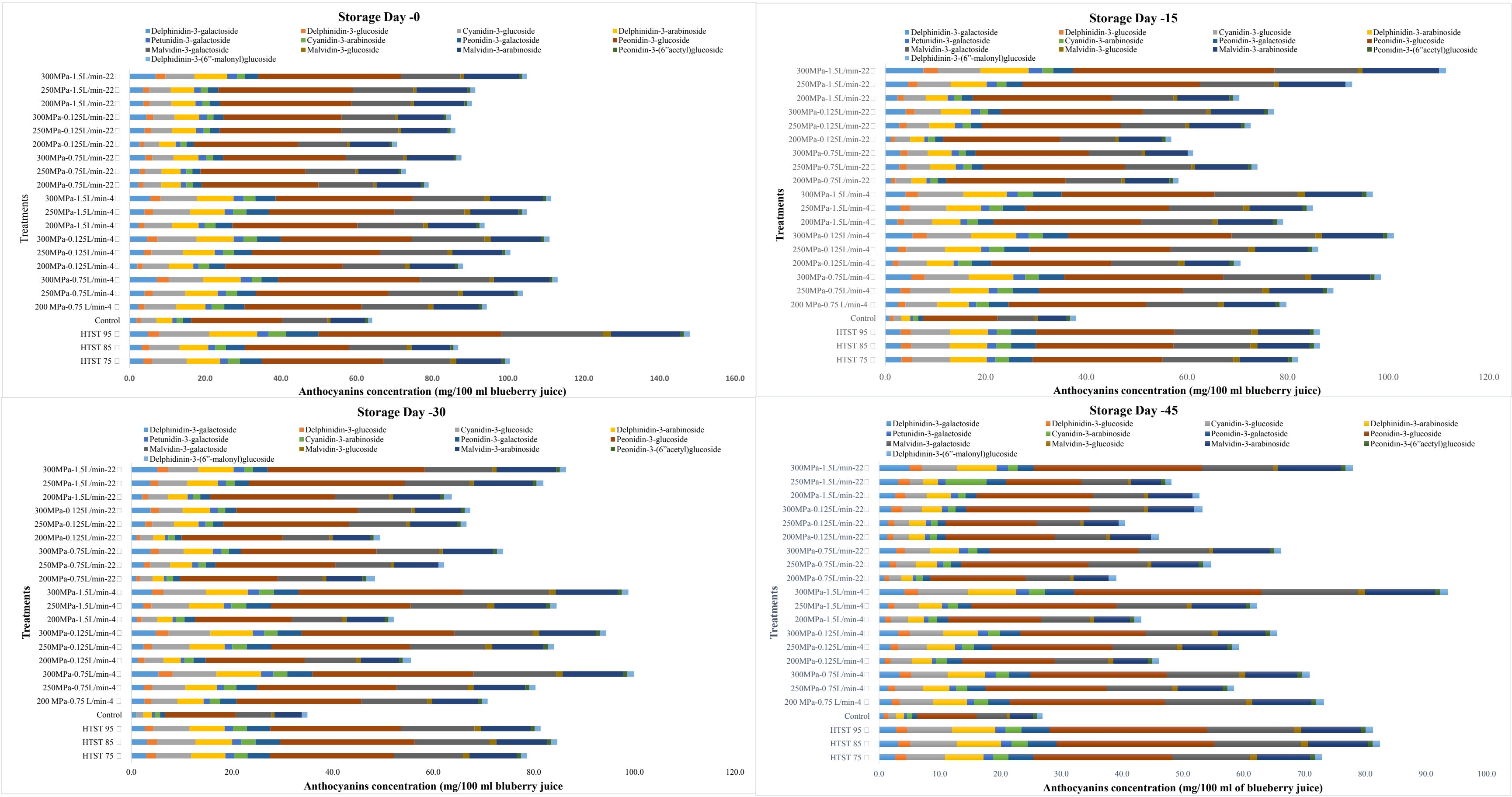

Figure S1. Anthocyanins concentration of various treated blueberry juice during cold storage at 4 °C.
